# GIS-based risk assessment of flood disaster in the Lijiang River Basin

**DOI:** 10.1038/s41598-023-32829-5

**Published:** 2023-04-15

**Authors:** Li Ziwei, Tang Xiangling, Li Liju, Chu Yanqi, Wang Xingming, Yang Dishan

**Affiliations:** Guilin University of Technology, Guilin, 541000 China

**Keywords:** Climate sciences, Ecology, Natural hazards

## Abstract

This study is designed to provide a scientific reference for the establishment of rainstorm and flood disaster prevention system in Guilin region and improve the risk assessment of rainstorm and flood disasters. To realize the goal, a flood risk evaluation model is established by weight analysis methods including the entropy weight method and the analytic hierarchy process from 3 aspects, i.e., risk of disaster causing factors, sensitivity of disaster-pregnant environment and vulnerability of disaster bearing body. For the model, the daily precipitation 1980–2020 of 6 representative national meteorological stations in the Lijiang River Basin was used as reference data of disaster causing factors; six indicators, i.e., NDVI, river network density, geological hazard, slope, slope aspect and terrain undulation were selected as the sensitivity of disaster-pregnant environment; NPP, potential of farmland production, and population density were taken as the criteria for determining the vulnerability of disaster bearing capacity. Meanwhile, ArcGIS was used for analysis and calculation to complete the risk assessment of flood disaster in Lijiang River Basin, Guangxi. The results indicate that: (1) the hazard level of flood disaster causing factors in Lijiang River Basin shows a decreasing distribution pattern from north to south, and high-risk areas cover 3108.47 km^2^, accounting for 21.29%; (2) the stability grade of disaster-pregnant environment shows a decreasing trend from the surrounding mountains to the plains, and the low-stability and lower-stability areas are mostly found in the low-lying areas around Lijiang River, with an area of 4218.63 km^2^, accounting for 28.69%; (3) the vulnerability of the disaster bearing body is generally at a low level, and the areas with high level cover 246.96 km^2^, accounting for only 1.69%; (4) under the combined effect of the above factors, the northern part of Guilin City in the Lijiang River Basin has a high risk of flood disaster.

## Introduction

Natural and technological disasters are also known as Natech events. One of the main natural disasters that cause Natech events is flood disaster^1^. With the climate warming and the rapid growth of national economy, the economic losses caused by flood disasters are becoming more and more serious. According to statistics, worldwide, there are as many as 20,000 people died and more than 25 million persons displaced by flood disasters every year^2^. Risk analysis and assessment of flood disasters is an important part of flood disaster management^3^, and climate change brings great challenges to flood risk assessment^4^, which is important not only in theoretical research but also in the practice of effective flood loss mitigation.

Various factors need to be taken into overall consideration for flood risk assessment, including basin characteristics, meteorological characteristics and regional characteristics. Due to the scarcity of information, the complexity of influencing factors, and the diverse probability distribution, there is a great uncertainty in flood disaster assessment^5^. There are three main methods for flood risk assessment: the first one is based on historical data^6,7^, the second one is based on systematic indicators^8,9^, and the third one is based on scenario analysis^10,11^. The first method is simple to operate and more objective, but it is difficult to obtain data, requiring data with high spatial and temporal resolution, and is difficult to adapt to small scales^12^. For the second method, Hu et al. assessed the regional flood risk by establishing a multi-indicator assessment system^8,13^, but this method has the shortcoming of high subjectivity in determining indicator weight. The three methods mentioned above are achieved by applying mathematical theories such as AHP^8,14^, gray clustering method^15^, fuzzy mathematics^16^, and Bayesian networks^17^. Among them, the AHP method is an effective method of risk assessment as it does not require a prior probability estimation and it is based on multiple indicators for qualitative and quantitative analysis^18^_,_ and has been widely used in disaster risk assessment in recent years^19^. However, the AHP method is more subjective in determining weights, and the integrated subjective–objective weight method reduces the subjectivity in weight setting and improves the scientific nature of evaluation. The entropy weight method is one of the methods to determine objective weights, and Wu et al. combined the AHP method with the entropy weight method to evaluate the flood disaster risk in the Huaihe River basin in different periods^20^.

Guangxi is located in the coastal area of South China and in the subtropical monsoon climate zone with abundant water and heat conditions. Heavy rainfall may occur throughout the year under the influence of alternating westerly and tropical systems. Especially, in the past 10 years, many places in Guangxi experienced heavy to extraordinarily heavy rainfall processes, causing flood disasters, which had a significant impact on the safety of people's lives and economic property. The Lijiang River Basin is located in the central area of heavy rainfall in Guangxi, with complex hydrogeological conditions, and is one of the more serious basins in the flash flood-prone area of Guangxi^21^. Therefore, this study assessed the flood disaster risk of Lijiang River Basin, Guangxi by combining the AHP method and the entropy weight method, so as to provide useful theoretical significance for scientific response to flood disasters under global warming, and protecting the safety of people's lives and property.

## Overview of the study area

### Overview of Lijiang River basin

**Figure 1:**
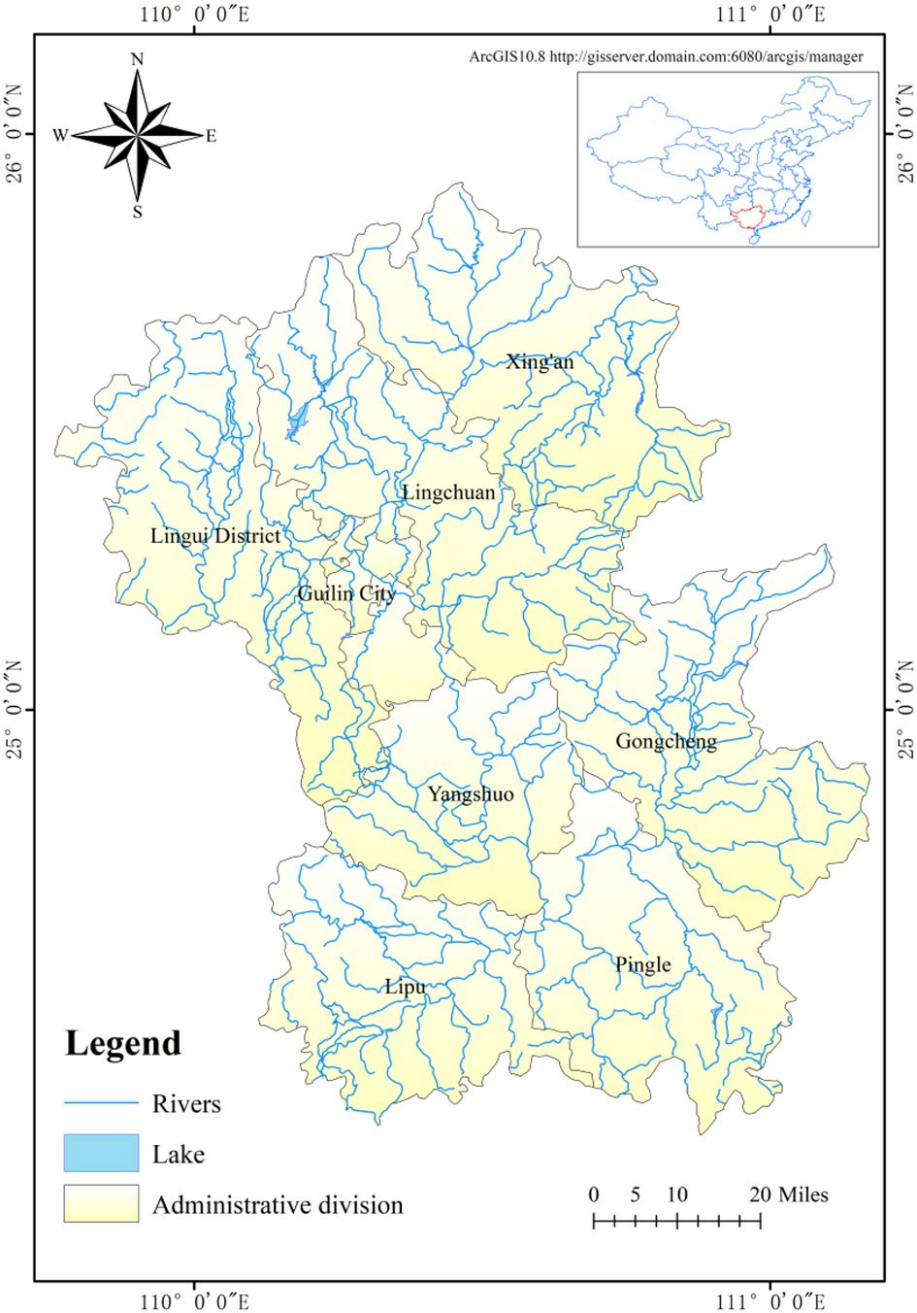
Water system of Lijiang River Basin.

Lijiang River, a West River system of the Pearl River basin, is located in the northeastern part of the Guangxi Zhuang Autonomous Region. Its source is located at the eastern foot of the Maoer Mountain, the main peak of Yuecheng Mountain, with an altitude of 2141.5 m, in Huajiang Town, Xing'an County, Guilin City. From north to south, it flows through Xing'an County, Lingchuan County, urban area of Guilin City, Yangshuo County, and Pingle County successively, with a total length about 229 km and an area about 5831 km^2^. Its main tributaries include Huangbai River, Ludong River, Chuanjiang River, Darong River, and Linghe River^22^. The study area covers the Lijiang River Basin areas of Guilin City, Xing'an County, Lingchuan County, Yangshuo County, Lipu County, Pingle County, and Gongcheng Yao Autonomous County, with an area of 14,597 km^2^ (Fig. 1).

### Hydrographic features

The average annual rainfall in the Lijiang River basin is 1941.5 mm, the maximum annual rainfall is 2460.7 mm, and the minimum annual rainfall is 1543.2 mm. Affected by the monsoon climate, the annual distribution of rainfall is very uneven. Rainfall is mainly concentrated in the first half of the year, with the rainy season from March to August, more rainfall from April to July, the peak rainfall from May to June, and the rainfall in May being the highest in the year. After September, the rainfall in a large area decreased, mostly local showers. From the geographical distribution of rainfall, the northern part of the basin is a high rainfall area, which gradually decreases to the south. The Huajiang, Chuanjiang, Guantian, Shangdong and Gaozhai areas in the upper reaches of the Lijiang River are one of the high value rainstorm areas. The average annual rainfall in the central area is 2600 mm, the maximum rainfall in three hours is 271.9 mm, the maximum rainfall in 24 h is 422 mm, and the maximum annual rainfall in the Huajiang River is 3500 mm, which is the main source of rainstorm causing the flood disaster in the Lijiang River. The average annual rainfall in Lingchuan Sanjie and Guilin is 1900 mm, and the rainfall in the basin is decreasing from northeast to southwest. According to the data of Darongjiang Hydrological Station, the annual runoff depth is more than 1600 mm. The spatial and temporal (mainly time) distribution of rainfall is extremely uneven. From the perspective of interannual changes, the maximum annual rainfall in Guilin is 2911 mm (1935), and the minimum is 1342 mm (1969), with a difference of 2.17 times. Due to the influence of the monsoon climate, the annual distribution of rainfall is very uneven. The rainfall from March to August accounts for 76% of the total rainfall of the whole year, of which the rainfall from April to June often accounts for more than 50% of the whole year, while the rainfall in autumn and winter only accounts for 24%.

The nonuniformity of rainfall determines the nonuniformity of runoff in the Lijiang River. Inter-annual variation of runoff: the measured annual average runoff of Lijiang River at the section of Guilin Hydrological Station is 40.3 × 10^8^ m^3^, measured maximum 56.3 × 10^8^ m^3^ (1968), the measured minimum value is 23.3 × 10^8^ m^3^, the difference is 2.4 times. The monthly runoff distribution within the year is similar to the annual distribution of rainfall in the basin. The runoff from March to August accounts for 77.5% of the whole year, of which the runoff from May to June accounts for 37.7%, which is the annual high value period, and the runoff from December to January of the next year accounts for 4.5%, which is the low value period. The difference between the high value period and the low value period is 8.4 times. The average annual flow is 128 m^3^/s, the average annual water level is 141.43 m, and the drainage area is 2860 km^2^.

## Data source and study method

### Data source

**Figure 2:**
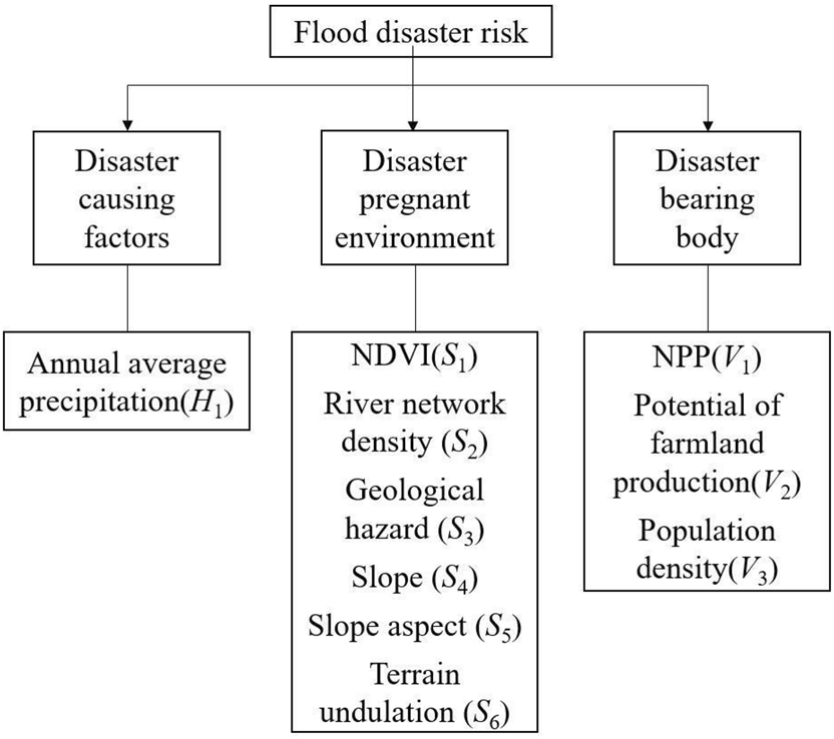
Flood Risk Assessment Indicator System.

According to the principle of flood risk assessment, the principle for indicator selection, and evaluation method, a flood risk assessment indicator system was established for the Lijiang River Basin with reference to the flood risk indicator system established by Weiguo et al.^24^: rainfall is the main hazard indicator of disaster causing factors, for which one indicator, i.e., annual average precipitation, was selected. Six main indicators, i.e., NDVI, river network density, geological hazard, slope, slope aspect, and terrain undulation, were selected for the sensitivity of disaster-pregnant environment. Three indicators, i.e., NPP, potential of farmland production, and population density, were selected for the vulnerability of the disaster bearing body (Fig. 2). The annual average precipitation data of each county were obtained from the Guilin Meteorological Service; the 12.5 m resolution DEM data of Guangxi Province were obtained from the Geospatial Data Cloud; the NDVI indicator data, population density data, Chinese NPP data, and Chinese farmland production potential data were obtained from the Resource and Environmental Science and Data Center under the Chinese Academy of Sciences; the data of geological hazard sites in the study area were obtained from the First Geological Team of Guangxi Zhuang Autonomous Region.

### Disaster risk assessment model

The disaster risk assessment model proposed by Peijun et al.^25^ is used to analyze the flood disaster risk, and the assessment formula is as follows:

1 $$R\left( {\text{x}} \right) = w_{H} H\left( x \right) \times w_{s} S\left( x \right) \times w_{V} V\left( x \right)$$

In Eq. (1), $R\left(x\right)$ is the flood disaster risk indicator, $H\left(x\right)$ is the hazard level of the flood disaster causing factors, $S\left(x\right)$ is the sensitivity of the flood disaster-pregnant environment, and $V\left(x\right)$ is the vulnerability of the disaster bearing body. Three of these factors can be calculated from Eqs. (2–4)^19^.

Hazard indicator of the disaster causing factors:

2 $$H\left( x \right) = \mathop \sum \limits_{j = 1}^{i} \left[ {w_{j} \times H_{ji} \left( x \right)} \right]$$

Sensitivity indicator of disaster-pregnant environment:

3 $$S\left( x \right) = \mathop \sum \limits_{j = 1}^{i} \left[ {w_{j} \times S_{ji} \left( x \right)} \right]$$

Vulnerability indicator of disaster bearing body:

4 $$V\left( x \right) = \mathop \sum \limits_{j = 1}^{i} \left[ {w_{j} \times V_{ji} \left( x \right)} \right]$$

where *H*_*ji*_(x), *S*_*ji*_(x), *V*_*ji*_(x) are the standardized values of the indicators; the values of *H*(*x*), *S*(*x*), *V*(*x*) correspond to the hazard of disaster causing factors, the sensitivity of disaster-pregnant environment, and the vulnerability of disaster bearing body, respectively; *w*_*H*_, *w*_*S*_, *w*_*V*_, and *w*_*j*_ are the weights of the assessment factors.

### AHP entropy weight method

For a certain indicator, the entropy value is used to determine its dispersion degree. The smaller the information entropy value, the greater the dispersion degree of the indicator, and the greater the weight of the indicator. Therefore, the information entropy is used to calculate the weight of each indicator, which provides the basis for the comprehensive evaluation of multiple indicators.AHP is a flexible and practical multi-criteria decision making method with simple principles and a rigorous mathematical basis, proposed by Saaty in the early 1970s^26^. It breaks down a complex problem into constituent factors and forms a hierarchy by the dominant relationship, and then uses pairwise comparisons to derive a scale of relative importance for decision alternatives. Broadly speaking, it can be carried out in the following four steps: (1) establishing hierarchical structure, (2) structuring all judgment matrices in each level, (3) ranking order and testing the uniformity, (4) ranking overall order and testing the uniformity^27,37–39^.

In order to determine the relative importance of each evaluation factor, the author conducted a questionnaire survey on a number of experts and scholars in different disciplines such as ecology, meteorology, geography, economics, sociology and geology (30 questionnaires were sent out and 28 were returned. The questionnaire uses fuzzy scoring criteria to divide the importance of indicators at all levels into 5 levels: very important, important, general important, unimportant and very unimportant). And according to the flood risk factors in the study area, the importance of the flood disaster assessment index system factors in the relevant references is selected and compared. Through sorting out the original effective data, the computer is used to process according to the above methods, and the weight value of each assessment index and the position of each factor are obtained. The calculation results of evaluation factor weight are as follows (Table 1).

**Table 1:** Weight distribution and sorting.

Total target layer	Weight	Comprehensive evaluation layer	Weight	Factor evaluation layer	Weight	Sort
Risk assessment of flood disaster in lijiang river basin	1	Disaster causing factors	0.185	Annual average precipitation	0.185	1
		Disaster pregnant environment	0.465	NDVI	0.040	8
				Density of river network	0.150	3
				Geologic hazard	0.038	9
				slope	0.079	6
				Aspect of slope	0.034	10
				Topographic relief	0.124	5
		Disaster bearing body	0.347	NPP	0.053	7
				Farmland production potential	0.128	4
				Population density	0.166	2

### Standardization processing

Since the data units are not uniform, the data need to be standardized by the following equation:

5 $$\alpha =0.1+\frac{I-{I}_{min}}{{I}_{max}-{I}_{min}}$$

6 $$\alpha =0.1+\frac{{I}_{min}-I}{{I}_{max}-{I}_{min}}$$

In Eqs. (5) and (6), α is the standardized data, I is the original series data, and *I*_*max*_* and I*_*min*_ are its maximum and minimum values, respectively, Eq. (5) applies to data items that are more likely to form high risk with larger values, and Eq. (6) applies to data items that are more difficult to form high risk with larger values^28^.

## Results and analysis

### Trend change of precipitation

**Figure 3:**
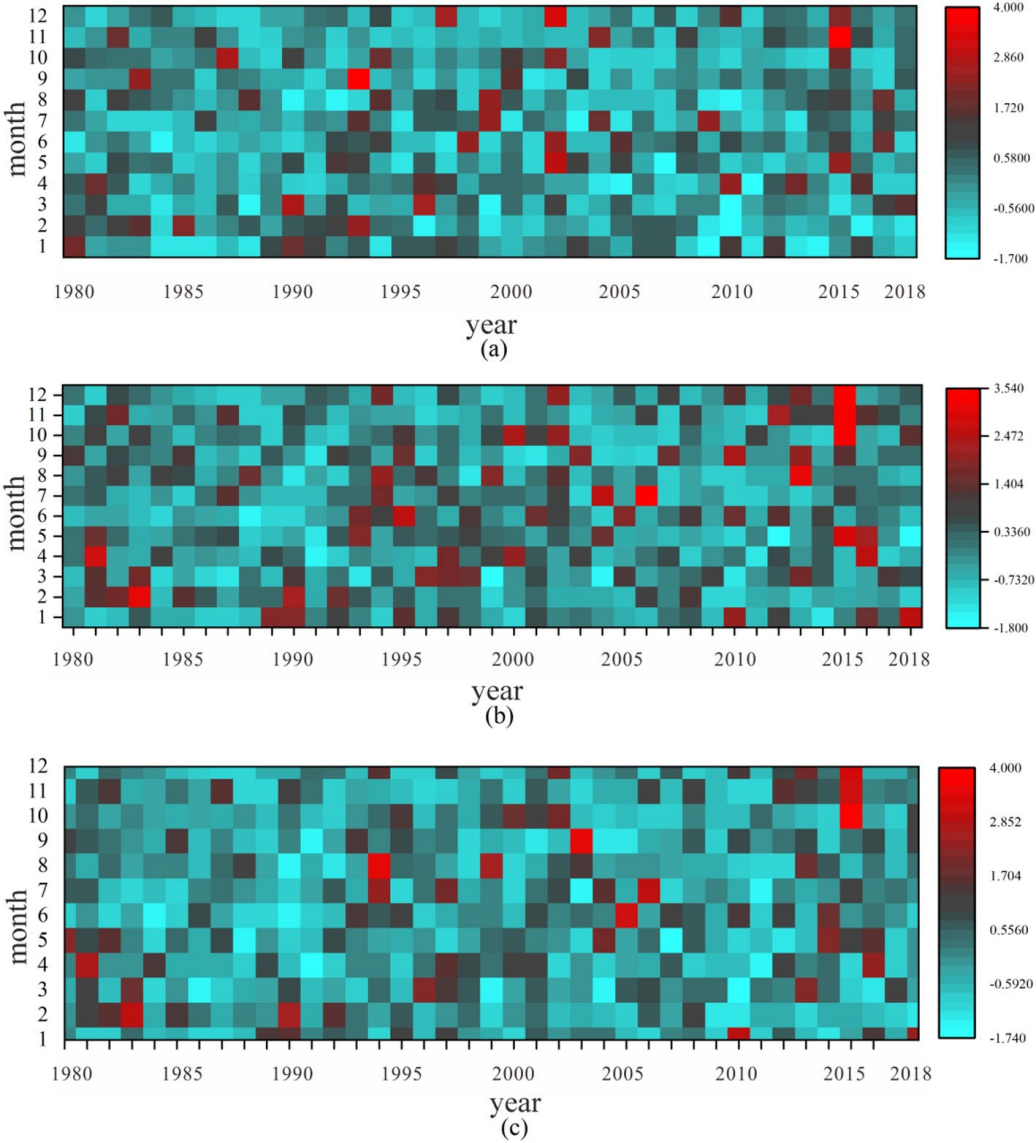

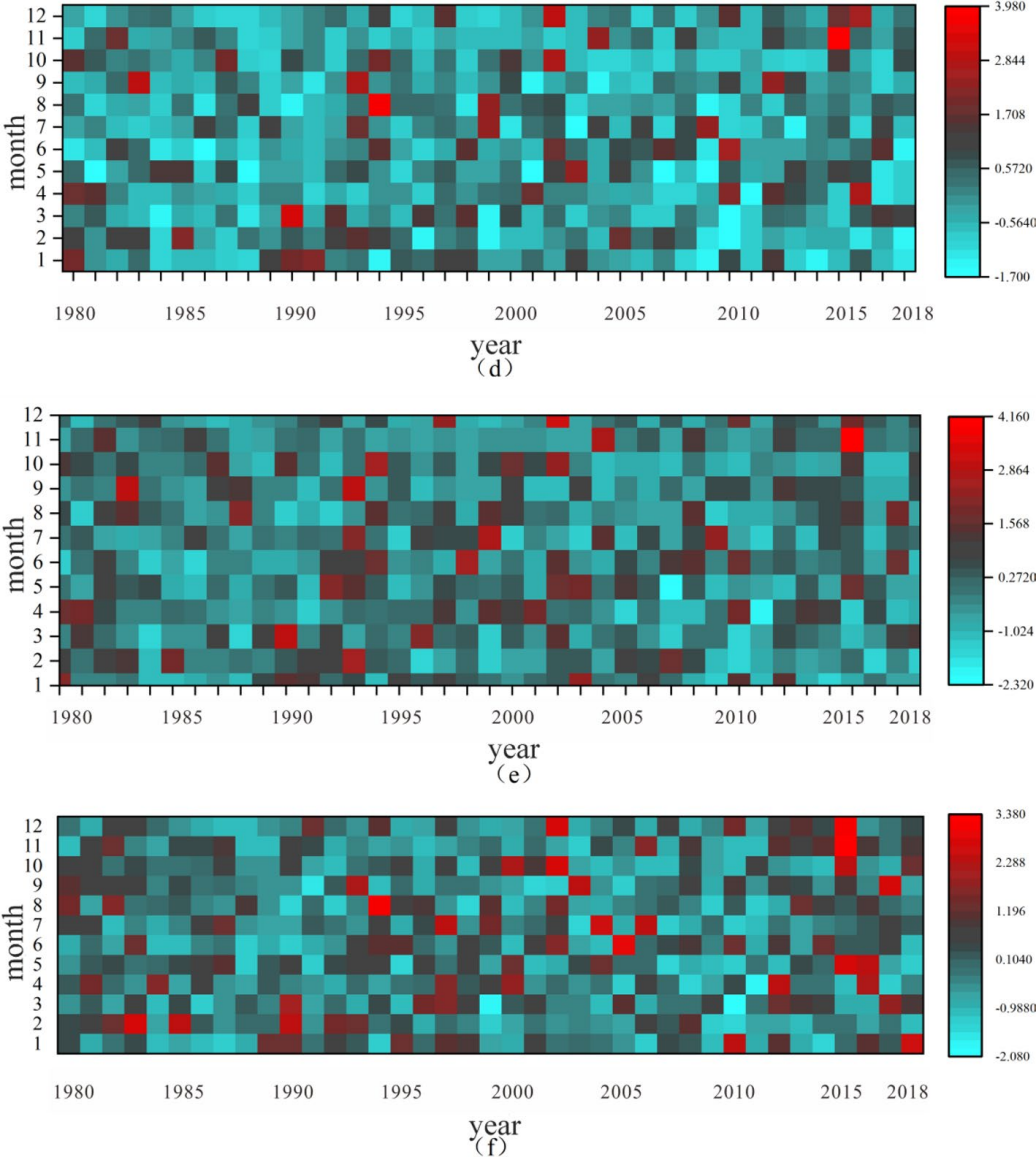
Heat map of interannual variation of precipitation in Lijiang River Basin (**a** Guilin, **b** Pingle, **c** Lipu, **d** Xing'an, **e** Lingchuan, **f** Yangshuo).

Highly developed river network and the spatial and temporal differences of precipitation in Lijiang River Basin are the main causes of flood disasters in the basin^17^. In order to better assess the flood risk, the 20–20 h precipitation of six counties in the Lijiang River Basin, Guilin, Pingle, Lipu, Xing'an, Lingchuan and Yangshuo, was analyzed for the period 1980–2018. According to the heat map analysis of interannual variation of precipitation in the Lijiang River Basin (see Fig. 3), the intra-annual distribution of precipitation at various stations in the Lijiang River Basin is extremely uneven, and the intra-annual distribution of precipitation varies greatly at different stations and in different years^29^, which is not conducive to the use of water resources in the basin; storm floods are frequent in the Lijiang River Basin, and 12 large storm floods have occurred so far after 1949, which are characterized by high intensity, long duration, and wide area. Among them, Guilin, Lipu and Yangshuo counties have greater precipitation and are more prone to storm flood disasters than the other three counties.

### Hazard of flood disaster causing factors

**Figure 4:**
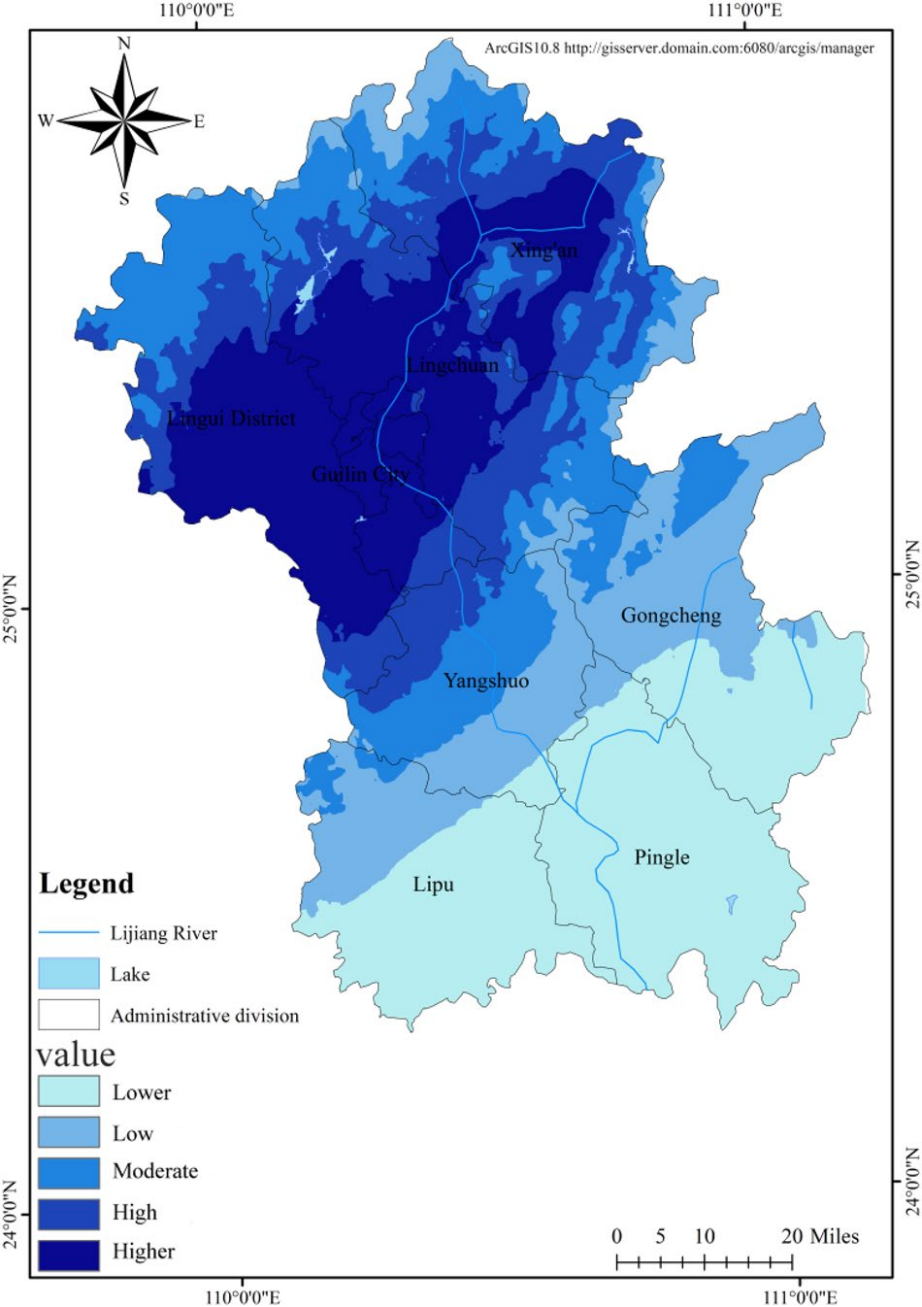
Spatial distribution pattern of hazard factors of flood disaster in Lijiang River Basin.

Rainfall is the inducing factor for flood disasters in the Lijiang River Basin. The monthly data of precipitation at meteorological stations in Guilin City, Xing'an County, Lingchuan County, Yangshuo County, Lipu County, Pingle County and Gongcheng Yao Autonomous County were collected and the annual average precipitation from 1980 to 2020 was calculated as the hazard indicator of disaster causing factors. According to the natural breaks classification method, the disaster-causing hazard indicator was divided into five levels: danger, high risk, moderate risk, low risk, security^30^, and the spatial distribution for the hazard of disaster causing factors in the Lijiang River Basin was obtained (Fig. 4). Driven by rainfall, the hazard level of disaster causing factors in the Lijiang River Basin shows a decreasing distribution pattern from north to south, as evidenced by frequent rainstorms in the upper reaches of Lijiang River, north of Guilin City and few rainstorms in South Guilin (Table 2). Higher areas cover 3,108.47 km^2^, accounting for 21.29%, and are concentrated in the middle and lower part of Lingui County, the municipal district of Guilin, central Lingchuan County and central Xing'an County. Lower areas (27.24%) and low areas (17.24%) cover 6 491.34 km^2^_,_ accounting for a large proportion, and are mainly distributed in Lipu County, Pingle County, Gongcheng Yao Autonomous County and southern Yangshuo County.

**Table 2:** Hazard level and influence range of disaster causing factors.

Level	Area (km^2^)	Ratio (%)
Lower	3975.48	27.24
Low	2515.86	17.24
Moderate	2675.23	18.33
High	2322.09	15.91
Higher	3108.47	21.29

### Stability of flood disaster-pregnant environment

**Figure 5:**
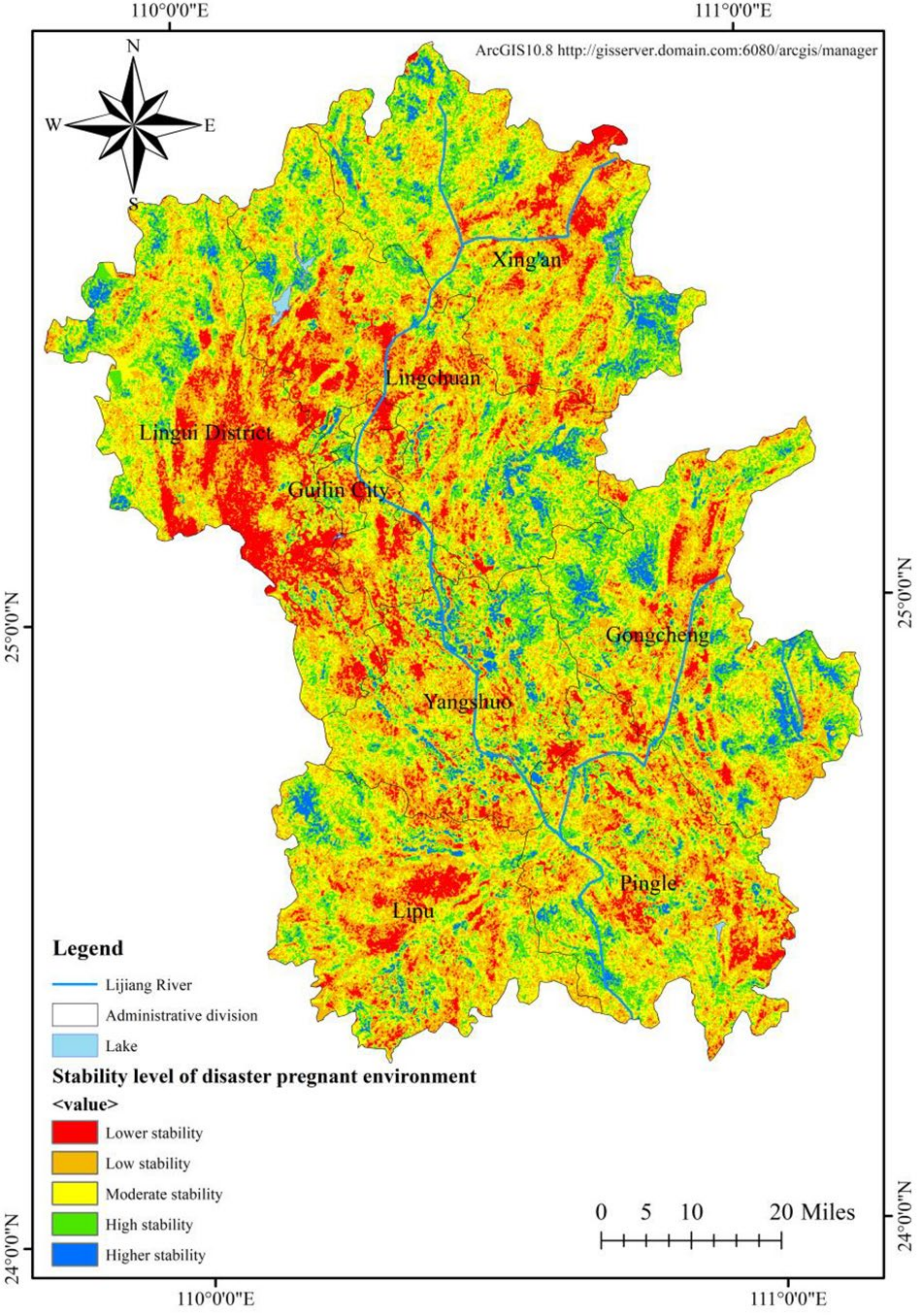
Distribution pattern of flood disaster-pregnant environment stability in Lijiang River Basin.

The spatial distribution of the stability of the flood disaster-pregnant environment in the Lijiang River Basin is shown in Fig. 5. According to the components of the stability of flood disaster-pregnant environment in the Lijiang River Basin as previously analyzed, six indicators, i.e., NDVI, river network density, geological hazard, slope, slope aspect, and terrain undulation, were used for analysis, and the stability grade of disaster-pregnant environment was negatively correlated with river network density, geological hazard, slope, slope aspect and terrain undulation, and positively correlated with NDVI. The stability grade of flood disaster-pregnant environment in the Lijiang River Basin shows a general trend of decreasing from the surrounding mountainous areas to the plains, as evidenced by high stability in the mountainous areas in the northern part of the basin, the southern part of Xing'an County and the intersection of Yangshuo, Lingchuan and Gongcheng counties, which are steep, of high topographic relief and of high altitude; and low stability in central Lingui County, southern Lingchuan County, the municipal district of Guilin City, central Lipu County and parts of Pingle County, most of which have slope less than 5°, elevation difference less than 60 m, dense river network and low vegetation coverage. Low stability and lower stability areas mostly exist in the low-lying areas around Lijiang River, with an area up to 4218.63 km^2^, accounting for 28.69%. The statistics of area for the stability grade of disaster-pregnant environment are shown in Table 3.

**Table 3:** Stability grade of disaster-pregnant environment and its influence range.

Grade	Area (km^2^)	Ratio (%)
Higher	2099.87	14.28
High	3983.51	27.09
Moderate	4400.79	29.93
Low	2981.65	20.28
Lower	1236.98	8.41

### Vulnerability of flood disaster bearing body

**Figure 6:**
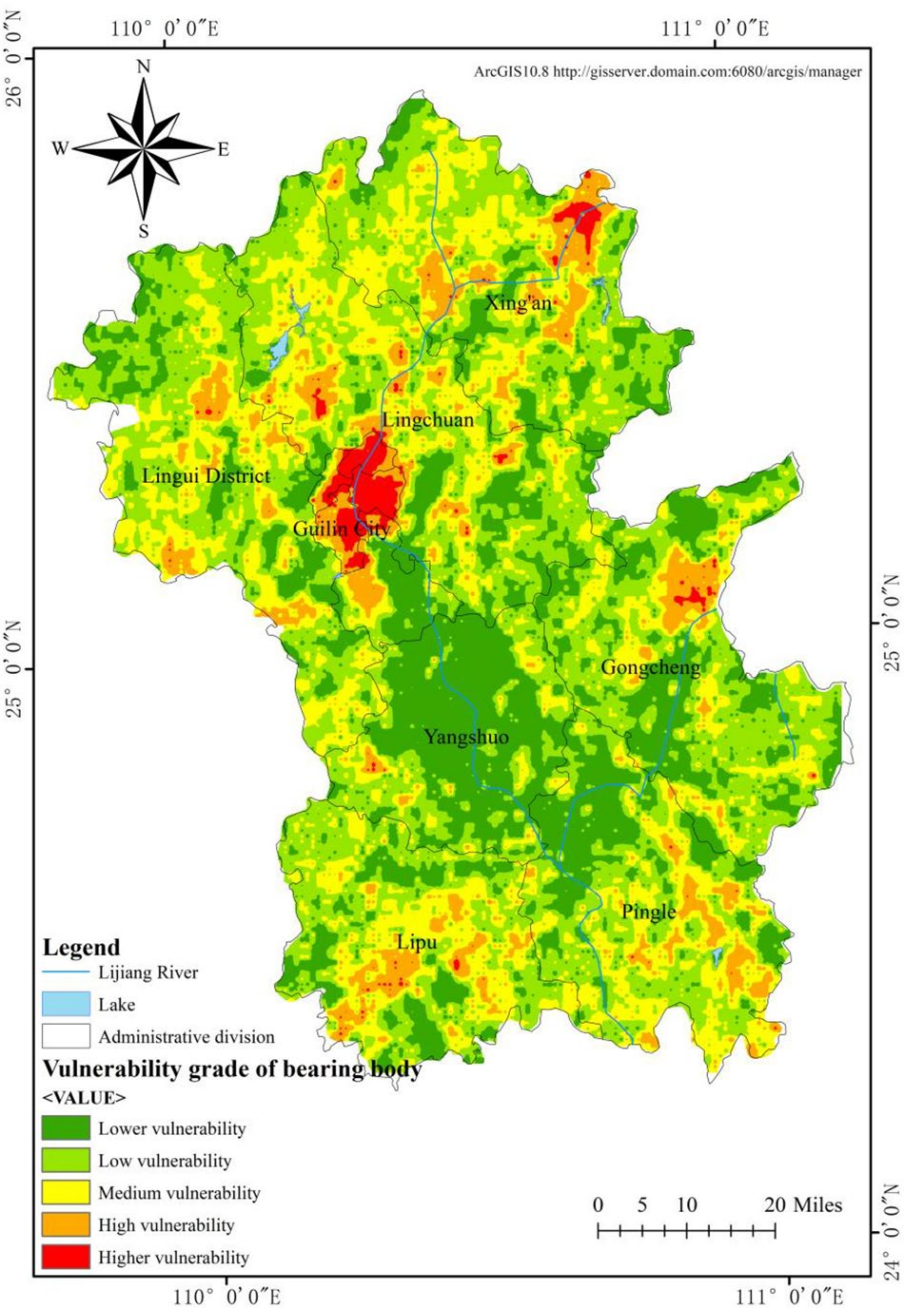
Spatial distribution pattern of vulnerability of flood disaster carriers in Lijiang River Basin.

Vulnerability of disaster bearing bodies is generally expressed using socioeconomic data^31,32^. Three indicators, i.e., population density, NPP, and potential of farmland production, were used to map the spatial distribution of the vulnerability of flood disaster bearing bodies in the Lijiang River Basin. As shown in Fig. 6, the vulnerability level of disaster bearing body in the Lijiang River Basin is generally at a low level, and the spatial distribution is higher in the central region than in other regions, with the highest vulnerability level in the municipal district of Guilin City. The areas with high vulnerability levels are concentrated in urban areas with population concentration and high NPP and farmland productivity. The statistical results of the areas of different levels are shown in Table 3. The areas with low vulnerability of disaster bearing bodies cover the largest area of 5725.76 km^2^, accounting for 39.29%, and the areas with high vulnerability of disaster bearing bodies cover 246.96 km^2^, accounting for only 1.69%. This reflects low economic level and small population in the Lijiang River Basin. Although the density and grade of flood control walls, flood diversion works, river training works, reservoirs, and other facilities in this basin are much less than those in areas with higher economic levels, the absolute losses will not increase as a result; instead, economically developed regions have a higher density and value of disaster bearing bodies, and the economic losses caused by floods of the same grade are greater^33^. The vulnerability level of bearing body and its influence range are shown in Table 4.

**Table 4:** Vulnerability level of bearing body and its influence range.

Level	Area (km^2^)	Ratio (%)
Lower	3652.11	25.06
Low	5725.76	39.29
Medium	3722.74	25.55
High	1225.53	8.41
Higher	246.96	1.69

### Food disaster risk

**Figure 7:**
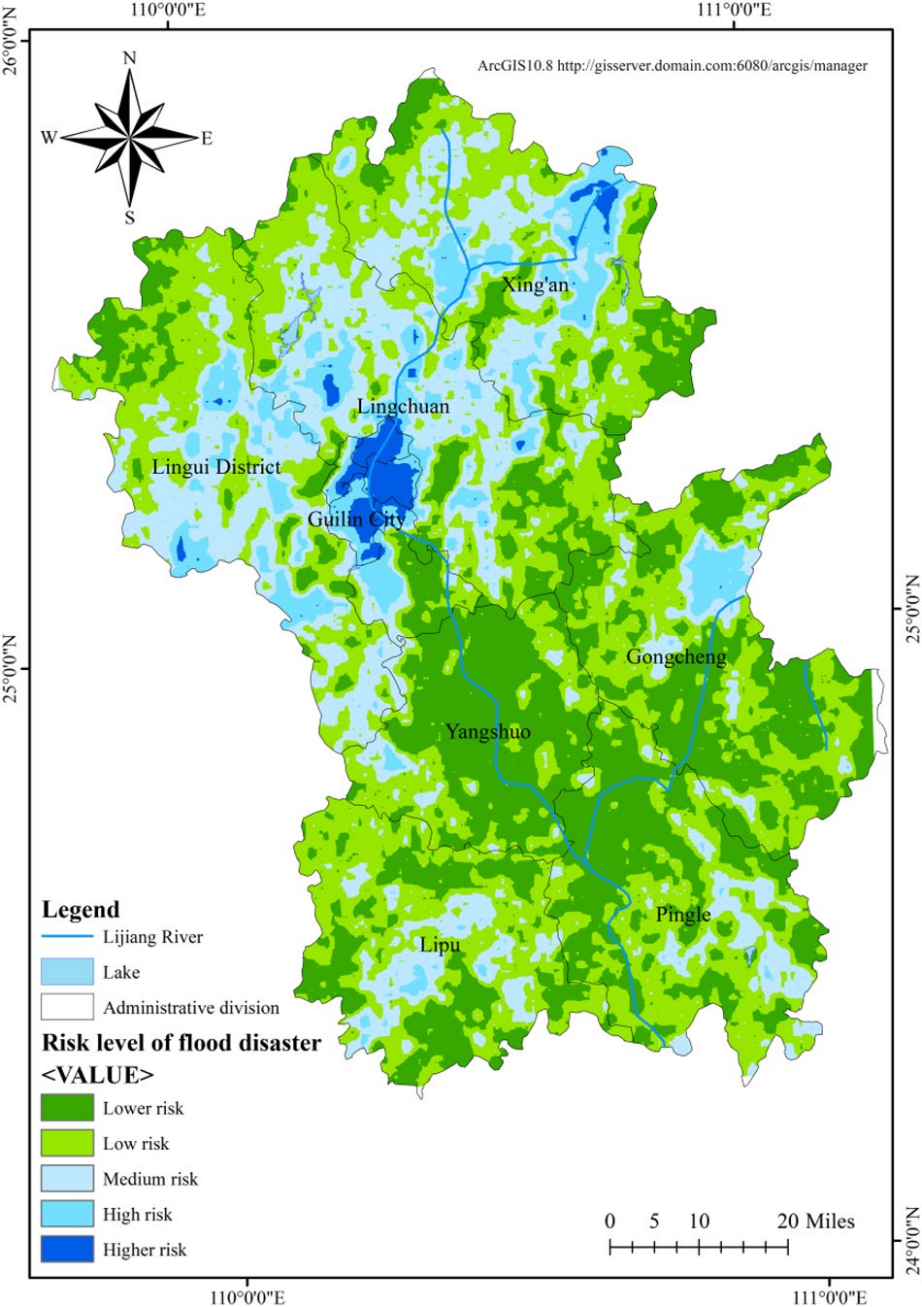
Risk level of flood disaster in Lijiang River Basin.

Based on the classification of the hazard of flood disaster causing factors, the stability of disaster-pregnant environment and the vulnerability level of the disaster bearing bodies^34,35^, the flood risk of the Lijiang River Basin was calculated and the flood risk evaluation map of the Lijiang River Basin was obtained (Fig. 7). It can be seen from Fig. 7 and Table 5 that the risk of flood disasters in the Lijiang River Basin is high. High-risk areas cover 232.01 km^2^ and are mostly concentrated in the municipal district of Guilin. Due to the topographic influence of the nearly east–west trending Yuecheng Mountain, the Haiyang Mountain to the east of Guilin, and the Tianping Mountain to the west, which constitute a blockage to the south-moving cold front, warm and humid air currents can easily accumulate in this area and form heavy rainfall^36^, while the municipal district of Guilin is also the more economically developed and most densely populated area in the Lijiang River Basin, with high vulnerability of disaster bearing body and low stability of disaster-pregnant environment, which can easily form flood disasters induced by heavy rainfall, in addition, there is also a small area distribution in western Xing'an County and Lingui District. High-risk areas cover 1109.60 km^2^, accounting for 7.53% of the total area, and are mainly distributed in the northern part of the basin, the northern part of Gongcheng Yao Autonomous County and the central part of Lipu County.

**Table 5:** Flood disaster risk level and its impact scope.

Level	Area (km^2^)	Ratio (%)
Lower	4458.75	30.27
Low	5653.01	38.38
Medium	3274.61	22.23
High	1109.60	7.53
Higher	232.01	1.58

## Results and discussion

Most areas in the study area are located in the rainstorm area in northern Guangxi, where the rainstorm is concentrated, frequent and intense, and the rainfall distribution is uneven in the year, mostly concentrated between March and August. The Lijiang River in the basin passes through Guilin City. Due to the impact of rainstorm, the Lijiang River section rises and falls sharply, making Guilin City under serious flood threat. The flood disaster in the study area has been serious in the past 60 years. See Table 6 for the overview of the flood disaster in the main disaster years.

**Table 6:** Overview of historical floods in Lijiang River basin.

Disaster year	Flood level (m)	Overview of flood disaster
June 27, 1949	147.23	Many districts and streets in Guilin were completely submerged. The disaster in Dongjiang District was serious, and the power supply of Zhishanyan Power Plant was cut off due to flooding. In this flood, 12 people were killed, and the area of flooded farmland was nearly half of the total urban area
June 6, 1952	147.43	Nearly 85% of the houses in Qixing District of Guilin City were flooded, 31 streets in urban and suburban areas were flooded, and 56.4% of the city's arable land was flooded
July 16–18, 1974	146.80	The northern and western parts of Guilin w0ere seriously flooded. 30 streets in the urban area were flooded, traffic was interrupted, and factories were shut down
July 5, 1992	147.11	47 streets in the urban area and 35 villages in the suburbs were flooded, with an area of 36.67 km^2^. The total economic loss of the city reached 51 million yuan
June 13–17, 1994	147.06	48 streets in the study area were flooded, and the traffic in Guilin was interrupted for more than 20 h, resulting in economic losses of 294.4 million yuan
June 18–24, 1998	147.70	The flooded area in Guilin urban area reached 25 km^2^, and the traffic was almost completely interrupted. The flood disaster caused a loss of 2.163 billion yuan
June 17–19, 2002	146.92	The flood control embankment has been built in Guilin urban area, but due to the flood flowing into the urban area from some river sections, and the insufficient flood drainage facilities in the urban area, the drainage is not smooth, which still caused relatively serious floods. The maximum submergence depth was 1.8 m and the maximum submergence time was 84 h, resulting in a total economic loss of 309.4 million yuan
July 3, 2009	147.55	More than 150,000 people were affected, mostly in the urban area of Guilin, and some houses collapsed
June 5, 2022	146.10	Some roads in the urban area of Guilin City were flooded, and roads in some districts were interrupted, resulting in traffic jam

**Figure 8:**
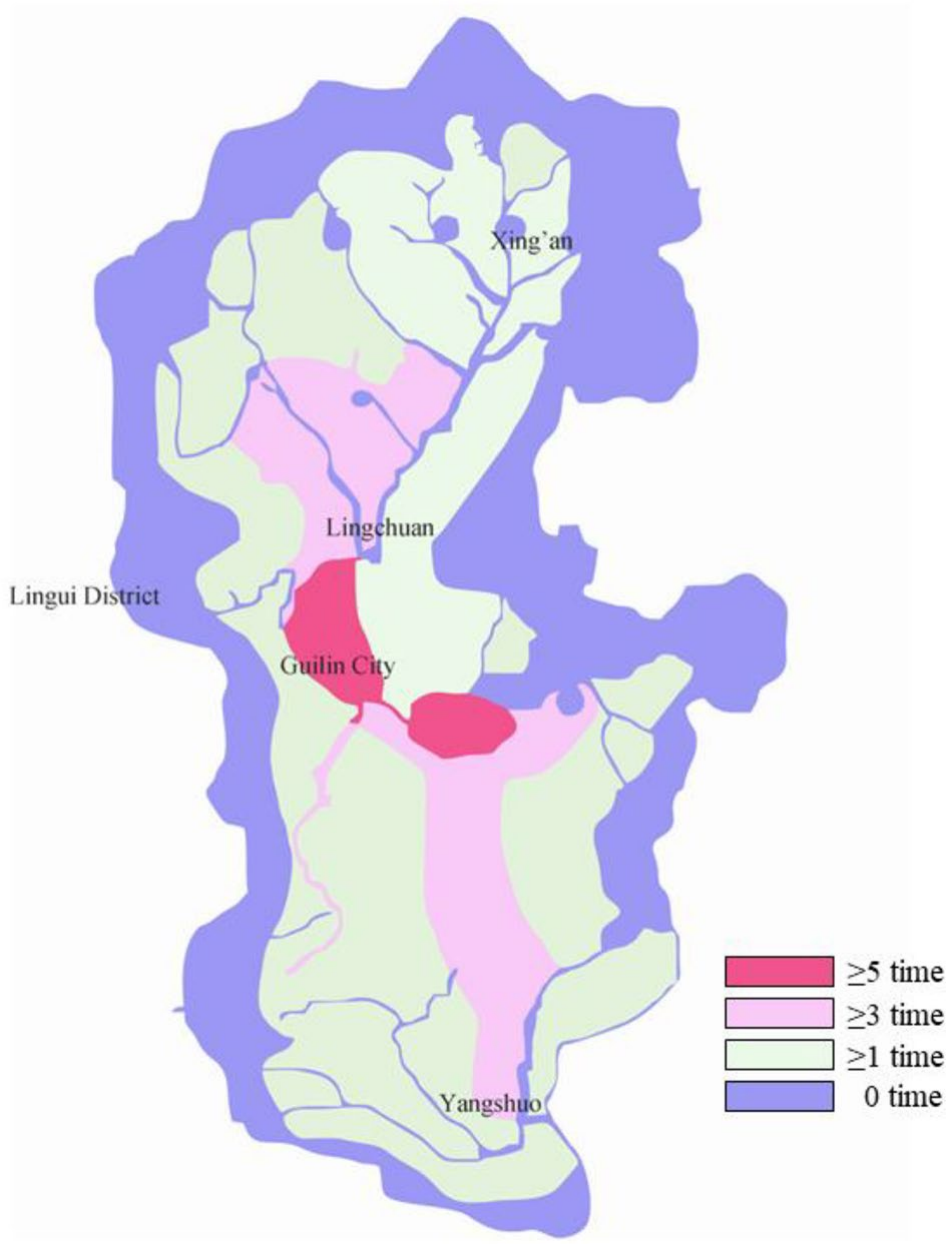
Frequency division of flood disaster in lijiang river basin in recent 60 years.

From the overall situation of the historical floods in the Lijiang River basin in the past 60 years, it is not difficult to see that Guilin and its suburbs are the most vulnerable areas to floods in the basin. The risk of flood disaster in the south of Guilin is relatively low, while the risk of flood disaster in the north of Guilin, such as Xiangshan District and Xiufeng District, is relatively high. Figure 8 shows the frequency division of flood disasters in the Lijiang River basin in the past 60 years. The upper reaches of the Lijiang River are the center of rainstorm in Guangxi, China, so flood disasters are concentrated on both sides of the river from Lingchuan to Yangshuo. Among them, the frequency of flood disaster risk in the urban area of Guilin and the southern part of Lingchuan is the highest. As long as the Lijiang River exceeds the warning water level, low-lying houses will flood and farmland will be flooded. The general situation of historical flood disasters in the Lijiang River basin in the past 60 years is roughly the same as the flood risk assessment results of the Lijiang River basin calculated by the model, with similar inundation range and high fitting degree. The model can better adapt to the Lijiang River basin and provide scientific decision-making for future flood risk prevention.

A comprehensive risk assessment model for flood disasters was developed taking three factors, namely, hazard of disaster causing factors, stability of disaster-pregnant environment and vulnerability of disaster bearing body, into overall consideration, to assess flood hazards in the Lijiang River Basin, and the following conclusions were drawn:

The hazard level of disaster causing factors in the Lijiang River Basin shows a decreasing distribution pattern from north to south, with high-risk areas mainly in the middle and lower part of Lingui County, Guilin City, central Lingchuan County and central Xing'an County. The reason for this is that the Yuecheng Mountain, Haiyang Mountain and Tianping Mountain block the south-moving cold front, and the warm and humid airflow tends to accumulate in the area, and the special topography leads to the formation of the rainy zone in the north of the basin; the overall stability level of disaster-pregnant environment shows a decreasing trend from the surrounding mountains to the plains, as evidenced by high stability in northern mountain area of the basin, southern Xing'an County and the intersection of Yangshuo, Lingchuan and Gongcheng counties, which are steep, of high topographic relief and high altitude; and low stability in central Lingui County, southern Lingchuan County, the municipal district of Guilin, central Lipu County and parts of Pingle County, most of which have slope less than 5°, elevation difference less than 60 m, dense river network and low vegetation coverage; the vulnerability level of the disaster-bearing body is generally low, and the high-vulnerability areas are concentrated in the municipal district of Guilin, where the population is concentrated and NPP and farmland productivity are high. The vulnerability level of the disaster-bearing bodies also reflects low population size and low economic level of the Lijiang River Basin.

## Conclusion

Based on GIS and flood disaster risk assessment model, this study found that the risk level of flood disaster causing factors in the Lijiang River basin was decreasing from north to south. The area of high-risk areas accounted for 21.29%, which was 3108.47 km^2^; The stability level of the disaster prone environment generally shows a decreasing trend from the surrounding mountains to the plains. Low stability and low stability areas mostly exist in low-lying areas around the Lijiang River, with an area of 4218.63 km^2^, accounting for 28.69%. The vulnerability level of hazard bearing bodies is generally at a low level, and the area with a high level is 246.96 km^2^, accounting for only 1.69%. Under the combined effects of the risk of disaster causing factors, the stability of disaster pregnant environment and the vulnerability of disaster bearing bodies, the risk of flood disasters in the northern part of Guilin City in the Lijiang River basin is relatively high, with a high risk area of 232.01 km^2^, accounting for 1.58%; The higher risk area is 1109.6 km^2^, accounting for 7.53%, which is distributed in the north of the study area.

The Lijiang River is the main river in the urban area of Guilin City, which has the characteristics of large valley slope. Due to the impact of rainstorm, the flood in the Lijiang River section rises and falls sharply, which also becomes the flood characteristics of Guilin City. In the past 60 years, Guilin has suffered from flood disasters on June 27, 1949, June 6, 1952, July 16, 1974, July 5, 1992, June 13, 1994, June 18, 1998, June 17, 2002, July 3, 2009, and June 5, 2022, with water levels reaching 147.23 m, 147.43 m, 146.80 m, 147.11 m, 147.06 m, 147.7 m, 146.92 m, and 146.10 m respectively. The floods occurred in the study area in the past 60 years have posed a threat to Guilin City. Among them, the flood in 1998 had a flow of 5,890 m^3^/s, resulting in the inundation of 47 streets in the urban area, especially in the north of Guilin. And the most serious flood occurred in the north of Guilin, which is basically consistent with the area calculated by the model.

## Data Availability

Resource and Environment Science and Data Center: https://www.resdc.cn. National Climatic Data Center: ftp://ftp.ncdc.noaa.gov/pub/data/noaa/isd-lite/. Geological Environment Monitoring Station of Guangxi Zhuang Autonomous Region: http://dzhj.dnr.gxzf.gov.cn/. National Meteorological Science Data Center: https://data.cma.cn/.
